# Hypogammaglobulinemia Class G Is Present in Compensated and Decompensated Patients with Propionate Defects, Independent of Their Nutritional Status

**DOI:** 10.3390/nu16111775

**Published:** 2024-06-05

**Authors:** Lizbeth Alejandra López-Mejía, Marcela Vela-Amieva, Sara Guillén-López, Daniela Mancera-Hernández, Isabel Ibarra-González, Edgar Alejandro Medina-Torres, Sara Elva Espinosa-Padilla, Cynthia Fernández-Lainez

**Affiliations:** 1 Laboratorio de Errores Innatos del Metabolismo y Tamiz, Instituto Nacional de Pediatría, Secretaría de Salud, Mexico City 04530, Mexico; 2 Servicio Social Facultad de Medicina, Benemérita Universidad de Puebla, Puebla Pue 72570, Mexico; 3 Unidad de Genética de la Nutrición, Instituto de Investigaciones Biomédicas, Universidad Nacional Autónoma de México (UNAM), Mexico City 04510, Mexico; 4 Laboratorio de Inmunodeficiencias, Instituto Nacional de Pediatría, Secretaría de Salud, Mexico City 04530, Mexico

**Keywords:** inborn errors of metabolism, methylmalonic acidemia, propionic acidemia, immunodeficiency, hypogammaglobulinemia, hypergammaglobulinemia

## Abstract

Propionate defects (PDs) mainly include methylmalonic (MMA) and propionic acidemia (PA) defects. Lifelong PD patients progress from the compensated to the decompensated stages, the latter of which are characterized by life-threatening acidemia and hyperammonemia crises. PD patients can suffer immunocompromise, especially during the decompensation stage. There is a significant gap in the research regarding the humoral immune response in PD patients. Here, we analyzed serum immunoglobulin concentrations and hemograms across compensated and decompensated stages in PD patients. Nutritional status and crisis triggers of decompensation were also explored. Twenty patients were studied, and 25 decompensation events (DE) and 8 compensation events (CE) were recorded. Compared with those in the CE group, the IgG levels in the DE group (513.4 ± 244.5 mg/dL) were significantly lower than those in the CE group (860.8 ± 456.5 mg/dL) (*p* < 0.0087). The mean hemoglobin concentration was significantly lower in the DE group (11.8 g/dL) than in the CE group (13.4 g/dL) (*p* < 0.05). The most frequent (48%) possible decompensation trigger factor was infection. Most of the events were registered in eutrophic patients (87.9%), despite which 65.2% and 50% of patients who experienced decompensated and compensated events, respectively, presented with hypogammaglobulinemia G. These findings provide evidence of the immunodeficiency of PD patients, independent of their nutritional status. We suggest that PD patients be managed as immunocompromised independently of their nutritional status or metabolic state (compensated or decompensated).

## 1. Introduction

**Figure 1:**
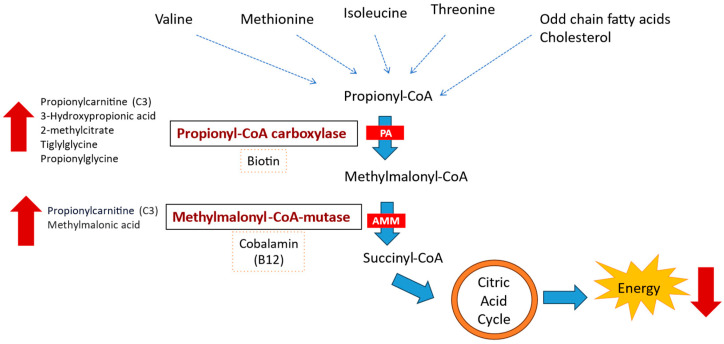
Metabolic pathway of propionate defects.

Organic acidemias (OAs) are a group of rare autosomal recessive disorders of intermediary metabolism that result in a systemic increase in organic acids [1,2,3]. Propionate defects (PDs) are among the most common OAs and are mainly represented by methylmalonic (MMA) and propionic acidemias (PAs). These PDs are caused by defects in the propionate catabolism pathway involving isoleucine, valine, threonine, methionine, odd-chain fatty acids, side-chain cholesterol, and anaerobic gut bacteria [4,5] (Figure 1). PD can present with acute, chronic, or intermittent symptoms and is typically diagnosed during acute metabolic decompensation that occurs after a trigger event. The trigger of an acute episode is catabolic stress induced by prolonged fasting, fever, acute trauma, increased intake of protein-rich foods, and infections. Furthermore, metabolic decompensation is related to high morbidity and mortality in PD patients [6,7,8]. Clinically, metabolic decompensation in PD patients is characterized by vomiting, feeding refusal, and drowsiness, followed by dehydration and neurological deterioration with hypotonia, seizures, irritability, or lethargy, which may lead to progressive coma and death [9,10]. The biochemical findings of metabolic decompensation crises are metabolic acidosis with an increased anion gap, hyperammonemia, hypoglycemia, ketoacidosis, hyperlactatemia, and hyperglycemia [7,8,9]. PD is considered a chronic, life-threatening, severely devastating condition with frequent episodes of metabolic decompensation that require repeated hospitalizations [2].

The interplay between infections and the immunological status of patients with IEMs must be fully explained [8]. Although immunological complications are becoming increasingly prevalent due to numerous inborn errors of metabolism (IEMs), their characteristics and mechanisms are poorly known [11]. Thus, the main goal of this study was to explore the serum levels of immunoglobulins and hematological parameters in PD patients during metabolic compensation and decompensation states.

## 2. Methods

### Patients

This study included 20 patients who were diagnosed with either MMA (11 out of 20, 55%) or PA (9 out of 20, 45%). Patients were recruited from the follow-up IEM cohort of the Inborn Errors of Metabolism and Screening Laboratory at the National Institute of Pediatrics in Mexico City. The participants were invited to participate, and written consent was obtained from their parents or guardians. As previously described, biochemical markers were quantified through tandem mass spectrometry, high-performance liquid chromatography, and gas chromatography–mass spectrometry [12]. Age, sex, and clinical data were recorded.

**Medical and nutritional treatment**. PA and MMA patients were treated according to the international guidelines of the Southeast Regional Genetics Network (SERN) [13]. Nutritional treatment consists of limiting intact protein to the age of dietary reference intake (DRI) to prevent the accumulation of toxic metabolites. For patients who tolerate less than 100% of the daily protein requirement for intact protein, amino acid-modified infant formula with iron is administered. Energy is managed to prevent catabolism. Levocarnitine is supplemented to maintain plasma-free carnitine in the normal range, and hydroxocobalamin is supplemented in patients with cobalamin deficiency. In decompensated patients with ammonia levels above 100 µmol/L, an emergency diet with restricted protein intake for a maximum of 48 h and high energy intake was administered [13].

**Samples, serum immunoglobulin quantification, and hemograms**. Blood samples were collected from patients in two stages, compensated or decompensated, to investigate the serum immunoglobulins and hematological parameters. Compensated stage events were defined as those presented by individuals who, at the time of the measurement of serum immunoglobulins, had a venous or arterial blood gas analysis with a pH in the normal range (7.32–7.42) and normal levels of ammonia (9–35 mmol/L). Decompensated stage events were defined as those presented by patients who, at the time of the measurement of serum immunoglobulins, had an abnormal venous or arterial blood gas analysis (pH < 7.32 or pH > 7.42) or hyperammonemia (>35 mmol/L). Events in the compensated or decompensated stages were recorded for all patients. Patients receiving immunoglobulin replacement therapy and patients who received blood transfusions 48 h before the measurement of serum immunoglobulins were excluded from the study. It is essential to mention that more than one sample might be collected from each patient if it is found in the compensated or decompensate stage.

Serum immunoglobulins IgA, IgE, IgG, and IgM were quantified using the Siemens Healthineers^TM^ platform with Siemens BN ProSpec^TM^ equipment and commercial kits following the manufacturer’s instructions. Serum samples were obtained from peripheral blood collected in tubes with a coagulation activator and then centrifuged for 30 min at 1500 rpm at 4 °C. The sera were diluted with N-DILUENT^TM^ reagent at a 1:20 ratio for IgA, IgM, and IgE and a 1:400 ratio for IgG. Calibration curves were constructed using serial dilutions of the N-PROT SL^TM^ standard reagent for each immunoglobulin and automated dilutions. Standard curve readings and samples were then performed to obtain concentration values for each immunoglobulin, ensuring that sample readings were within the range of the standard curve. The analysis of any samples outside this range was repeated or adjusted following the manufacturer’s instructions. Serum immunoglobulin concentrations were classified as low, usual, or high according to the reference values reported by our institution’s central clinical laboratory according to age.

The hemogram included hemoglobin concentration (g/dL) and the total count of leukocytes, neutrophils, lymphocytes, monocytes, eosinophils, and platelets (10^3^/mm^3^) according to the standardized laboratory measurements of our institution.

**Trigger factor analysis**. The possible trigger factors responsible for decompensation were classified as infectious, diet transgression, or both. The results of bacterial, fungal, or viral cultures or analyses were recorded when they were performed. In all cases, a 24 h dietary recall was performed to determine whether there was a diet transgression.

**Nutritional status evaluation.** As nutritional status is a well-known factor that can modify the immune system, anthropometric measurements (weight and height) were performed for each patient who experienced compensated or decompensated events. The BMI Z score was calculated with Anthro^®^ software (Version 3.2.2.1, Geneva, Switzerland) for patients aged 0–5 years and AnthroPlus^®^ software (Version 1.0.4., Geneva, Switzerland) for children aged 5–19 years. Assessments were stratified according to the World Health Organization (WHO) classification. For children aged 0–5 years, they were considered underweight (<−2 SD), eutrophic (−2 to +2 SD), overweight (>2 SD), or obese (>3 SD). For 5- to 19-year-old patients, underweight was defined as (<−2 SD), eutrophic as (<2 to 1 SD), overweight as (>1 SD), and obese as (>2 SD). It is important to note that these nutritional assessments form part of our routine evaluations of our patients. On this line, the follow-up of patients between the diagnosis and time of enrollment consisted of periodical nutritional, medical, and biochemical evaluations that included a 24 h dietary recall, anthropometric measurements, biochemical tests, and quantification of amino acids and acylcarnitines. Based on these evaluations, diet or supplements were adjusted whenever needed.

**Literature review**. Three researchers (M. V-A, C.F-L, and D.M-H) systematically searched PubMed, Scopus, Web of Science, and the Cochrane Library, with a date limit from 1980 to 2023, using combinations of the following search terms: “immunoglobulins”, “immune response”, “immunodeficiency”, “IgG”, “IgE”, “IgA”, “IgM”, AND “propionate defects”, “methylmalonic acidemia”, “propionic acidemia”, and “organic acidurias”.

**Statistical analysis**. The normality of the data was calculated with the Shapiro–Wilk test. For normally distributed data, one-way ANOVA, mixed-effects analysis using Geisser–Greenhouse correction, and Dunnett’s multiple comparison tests were performed. Nonparametric distributed data were analyzed with the Mann–Whitney *U* test, followed by Dunn’s multiple comparisons adjustment test. All these statistical analyses were performed with GraphPad Prism ver. 10.1.1.

## 3. Results

Eleven males and nine females were studied; eleven of these patients had MMA, and nine had PA. The overall mean age at diagnosis was 24.5 months (0.43–206.3), and the mean age at study enrollment was 67 months (1–200); this detailed information is shown in Table S1. There were eight compensated events (MMA: five events and PA: three events), and 25 decompensated events (MMA: 9 events and PA: 16 events) were recorded. The mean age of the patients in the compensated stage was 10.1 years, that of the MMA patients was 11.4 years, and that of the PA patients was 5.2 years, while the mean age of the MMA patients in the decompensated stage was 3.5 years, that of the MMA patients was 2.9 years, and that of the PA patients was 3.8 years. The mean values of venous blood gas parameters and ammonia concentrations of both studied groups are shown in Table 1.

Hemograms revealed anemia in 22.2% of the decompensation events, whereas anemia was not detected in the patients with compensation events (Table 2).

**Figure 2:**
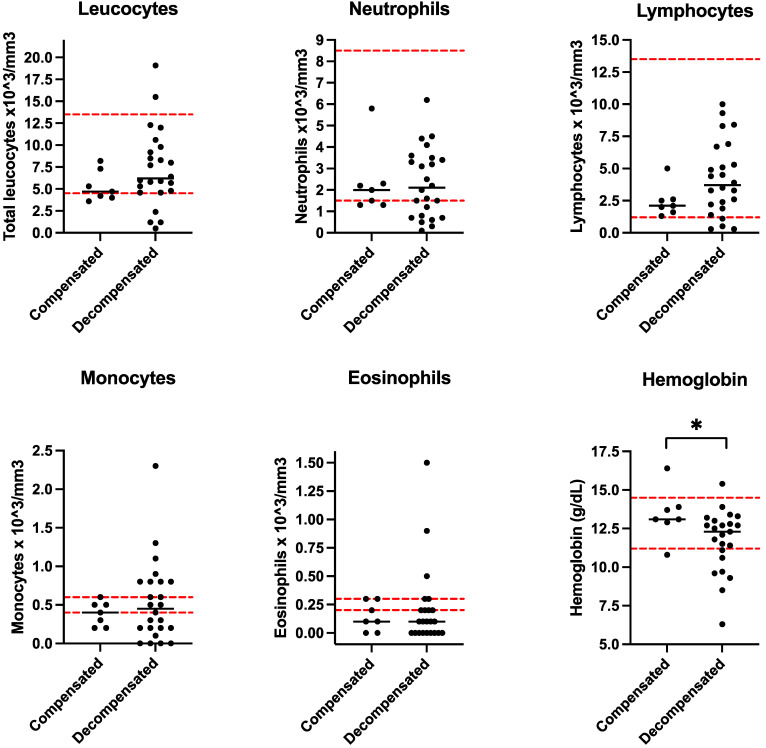
The total count of blood cells in patients with PD is divided into compensated and decompensated states. Dashed red lines are the standard reference interval of each parameter. * *p* < 0.05.

During compensation events, there was a significant difference in the mean monocyte count between PD patients (0.38 × 10^3^/mm^3^) and those who experienced decompensation events (0.51 × 10^3^/mm^3^) (*p* = 0.002). Additionally, the mean hemoglobin concentration was significantly lower in the patients who experienced decompensation events (11.8 g/dL) than in those who experienced compensation events (13.4 g/dL) (*p* < 0.05). There were no significant differences in the remaining blood cell counts between compensated and decompensated patients (Figure 2).

**Figure 3:**
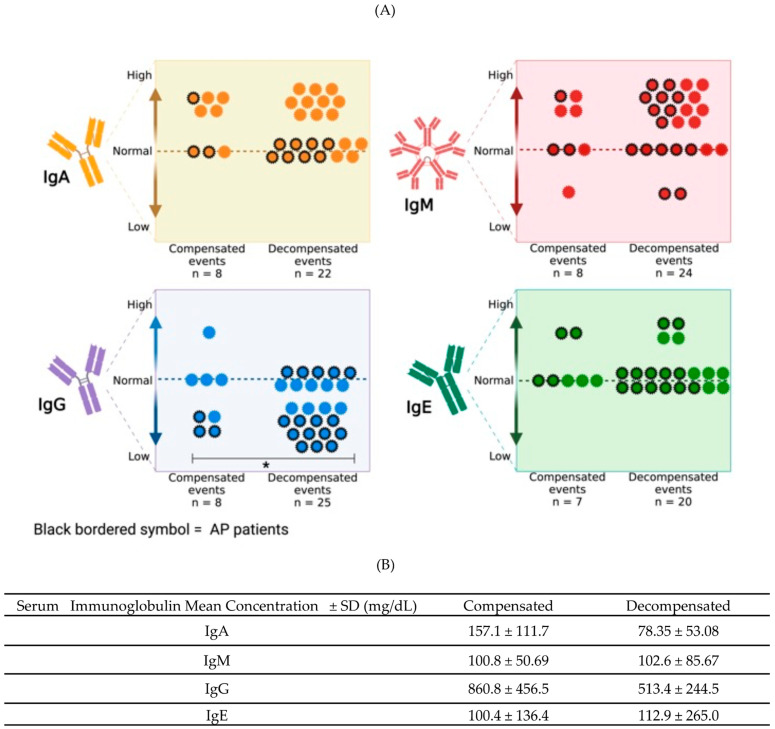
(**A**) Serum immunoglobulin levels of patients with propionate defects. Reference values according to age were obtained from our clinical laboratory. * IgG level was significantly lower in the decompensated stage compared with the compensated (*p* < 0.0087). (**B**) Mean ± SD of serum immunoglobulins concentrations.

In Figure 3, the immunoglobulin concentrations classified as low, normal, or high are presented and distributed by compensated or decompensated events. The mean level of IgG during decompensation events was 513.4 ± 244.5 mg/dL, which was significantly lower than that during compensation events, for which the mean value was 860.8 ± 456.5 mg/dL (*p* < 0.0087). No significant differences existed in the IgA, IgM, or IgE blood concentrations between compensated and decompensated patients (Figure 3B). A tendency toward elevated IgA and IgM in both compensation and decompensation events was observed.

The possible decompensation trigger factor was identified in only 15/25 events. Infectious factors, including viruses, bacteria, and combined pathogens, were the most frequent, and noncompliance with treatment was also identified in 3/25 (Table 3).

**Figure 4:**
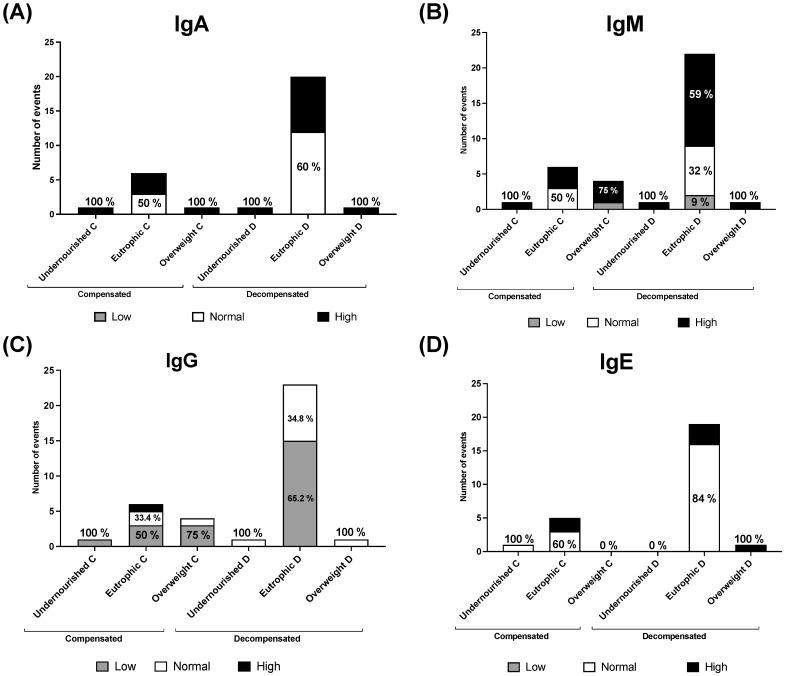
Percentage of immunoglobulin levels in PD patients during compensated and decompensated events divided by nutritional status. (**A**) Immunoglobulin A; (**B**) Immunoglobulin M; (**C**) Immunoglobulin G; (**D**) Immunoglobulin E. In the title of each bar, letter C indicates compensated; and letter D indicates decompensated.

The nutritional status of the studied patients was determined during every event. In total, 87.9% (29/33) of the events occurred in eutrophic patients, independent of whether they were compensated or decompensated. Later, nutritional status was studied by assessing compensated or decompensated events. In 75% (6/8) of compensated events, the individuals were eutrophic, while only 1/8 (12.5%) were undernourished or overweighed. In 92% of compensated events (23/25), the individuals were eutrophic, and 1/25 (4%) were undernourished or overweighed each. Furthermore, the serum immunoglobulin concentrations were evaluated according to the different nutritional statuses of PD patients. In eutrophic patients who experienced compensation events, we found that IgA levels were normal in 50% and high in 50% of patients. Moreover, IgA levels were normal for patients who experienced decompensation events (60%) and high (40%). Under the same scenario, IgM levels were also normal in 50% and high in 50% of patients during the compensated stage. This was different for patients in the decompensated stage; IgM levels were high at 59%, normal at 32%, and low at 9%. IgG levels were low in 50% of eutrophic patients during compensated events, normal in 33.4%, and high in 16.6%. During decompensation events, 65.2% of patients had low IgG levels, and 34.8% had normal IgG levels. Figure 4 shows the detailed comparison between immunoglobulin levels and the nutritional status of the patients during both compensated and decompensated events.

To further investigate the abnormalities found in immunoglobulins, we reviewed the literature and found that only five studies (1984–2020) reported abnormal serum immunoglobulin concentrations in PD patients (Table 4).

## 4. Discussion

There is a significant gap in the understanding of the humoral immune response of PD patients. In this study, we aimed to explore the immunological function of these individuals by analyzing the serum concentrations of immunoglobulins across two metabolic stages—compensated or decompensated—and by considering nutritional status and exploring probable crisis triggers; we found that infectious conditions are the most frequent. A previous study found that the absolute lymphocyte subset counts were low in 91% of PD patients, with CD4+ T-cell lymphopenia being the most common. We also found that 81% of patients presented a low CD4+/CD8+ T-cell ratio [19]. Our study corroborates the findings of other authors who reported that PD patients are prone to hypogammaglobulinemia class G [14,15,16,17,18]. Other authors have reported elevated IgG [16]; so, this topic remains controversial and deserves further study.

In addition to hypogammaglobulinemia G, our results suggested an immunological response pattern characterized by blood elevation of IgA, IgM, and IgE in compensated and decompensated PD patients. Nevertheless, none of those elevations were statistically significant (Figure 3). Other authors have found normal or low concentrations of IgA, IgM, and IgE [15,16,17,18].

While immunodeficiencies in patients with PD have been described by various authors as part of its natural history [16,20,21,22], to the best of our knowledge, this is the first study to correlate nutritional status with immunoglobulin levels in PD patients, regardless of whether they are in compensated or decompensated stages. Overall, it is well known that nutritional and immune status are correlated, i.e., undernourished patients are prone to low immunoglobulin levels [23], and overweight can provoke immunoglobulin alterations [24]. Remarkably, in this study, most of the patients were eutrophic (Figure 4); despite this, in eutrophic PD patients, we observed a greater percentage of hypogammaglobulinemia G in the decompensated stage (65.2%) than in the compensated stage (50%) (Figure 4C).

Hypergammaglobulinemia A was present in a slightly lower proportion of eutrophic patients in the decompensated stage (40%) than in compensated patients (Figure 4A). A greater proportion of high IgM was detected in eutrophic patients in the decompensated stage (59%) than in those in the compensated stage (50%) (Figure 4B). The main function of circulating immunoglobulins IgA, IgM, and IgG is to protect the individual against invasion by foreign pathogens [25]. IgG plays an important role in neutralization, opsonization, and sensitization to kill NK cells and activate the complement system. Thus, low immunoglobulin levels could be related to several immune dysfunctions [26]. Overall, high levels of immunoglobulins can also be related to an inflammatory state [27,28].

Furthermore, our study provides evidence of the high frequency (48%) of infection as a trigger factor of metabolic decompensation. It is important to note that among the pathogenic infectious agents, we found mainly opportunistic germs such as *Pseudomonas* and *Staphylococcus* or cytomegalovirus (Table 3). Thus, our results suggest that PD patients should be considered a population at risk for opportunistic infections. Opportunistic infections are pathogens that take advantage of the host’s weakened immune system [29,30]. The conditions of a host that predisposes him or her to an opportunistic infection are well known and include profound and prolonged neutropenia, clinical instability or significant medical comorbidities, cancer and chemotherapy, primary immunodeficiency, cystic fibrosis, catheters, prolonged use of steroids or immunosuppressive drugs, HIV infection, prolonged hospitalizations, and malnutrition, among others [29]. Based on this evidence, we propose that MMA or PA must be considered a condition predisposing patients to opportunistic infections, and these patients must be considered and managed for immunodeficiency.

We found a greater number of monocytes in PD patients in the decompensated stage than in those in the compensated stage; this was different for the hemoglobin concentration, which was significantly lower in the decompensated patients. (Figure 2). These findings corroborate the fact that PD patients are prone to chronic hematological dysfunction [7], especially patients who are decompensated.

Taken together, these findings suggest immunological dysfunction in the studied patients. The mechanisms underlying our findings may be related to the toxicity to the immune system caused by the metabolites that accumulate in these disorders; however, further studies are required to elucidate these mechanisms.

One limitation of this study is that due to the low prevalence of AP and AMM, we considered the affected patients to constitute the same group; these defects are related to the initial metabolic pathway, some biomarkers, and the clinical picture; however, each one has its own genetic and metabolic characteristics [2]. Another limitation is the small number of studied patients due to the low frequency of these disorders. Despite these limitations, our results indicate the need for this group of patients to undergo permanent immunological evaluation, both in the compensated and decompensated stages. We also propose the incorporation of immunoglobulin quantification in the current guidelines for diagnosing and managing PD patients [7].

## 5. Conclusions

In conclusion, the hypogammaglobulinemia G observed in the studied PD patients in both compensated and decompensated states, along with the frequent presence of opportunistic germs associated with metabolic decompensations, provides evidence of an immunodeficient condition in PD patients, independent of their nutritional status, and highlights the necessity of considering them immunocompromised individuals who need prophylactic measures such as gamma globulin and antibiotic administration.

## Figures and Tables

**Table 1:** Venous blood gas parameters and ammonia concentration in patients of compensated and decompensated events.

Event	pH	pCO_2_ mmHg	pO_2_ mmHg	HCO_3_ mEq/L	BEecf mL/dL	Lactate mmol/L	Anion Gap mEq/L	Ammonia mmol/L
Reference value	7.32–7.42	38–52	24–48	19–25	−5–+5	1–3.3	12 ± 2	9–35
Compensated	7.38	44.9	30.9	26.1	1.3	1.6	15.7	28.1
Decompensated	7.36	36.1	63.9	20.9	−6.3	3.7	15.6	104.3

**Table 2:** Hemogram results in PD patients during compensated and decompensated events.

Cytopenia	Compensated Stage (N = 8)	Decompensated Stage (N = 25)
Anemia	0/8 (0%)	4/25 (16%)
Leukopenia	3/8 (37.5%)	7/25 (28%)
Neutropenia	1/8 (12.5%)	6/25 (24%)
Lymphopenia	1/8 (12.5%)	7/25 (28%)
Thrombocytopenia	2/8 (25%)	7/25 (28%)
Bicytopenia (two affected cell types)	1/8 (12.5%)	5/25 (20%)
Pancytopenia (more than two affected cell types)	0/8 (0%)	3/25 (12%)

**Table 3:** Trigger factors accountable for the decompensated events in studied patients with PD (25 events).

Trigger Factor		Characteristics
Infectious 12/25 (48%)	Viral 3/12 (25%)	Cytomegalovirus (blood)
		Respiratory human metapneumovirus (blood)
		Adenovirus + syncytial respiratory virus (blood)
	Bacterial 3/12 (25%)	*Pseudomonas aeruginosa* (blood)
		*Staphylococcus* sp. penicillin-resistant (blood)
		*Pseudomonas aeruginosa* (urine)
	Viral + bacterial 2/12 (16.7%)	Respiratory OC-43 coronavirus + *Pseudomonas aeruginosa* (blood)
		Rhinoenterovirus + *Staphylococcus epidermidis* (blood)
	Not identified, but possibly infectious 4/12 (33.3%)	Fever (3 cases) Pneumonia (1)
Non-compliance to treatment 3/25 (12%)		
Not known or not identified 10/25 (40%)		

**Table 4:** Other studies that have reported abnormalities in immunoglobulins in PD patients.

Study	Disease (Number of Patients)	Type of Study	Under Compensated or Decompensated Stage (Number)	Immunoglobulin Results
[14]	MMA (4)	Case series	Not specified	Evaluation: IgG, IgM, IgA, IgE. Low IgG in two cases: 460 mg/dL (normal 630–1280 mg/dL) and 601 mg/dL (normal 610–1570 mg/dL). High IgE in two cases: 154 U/mL and 264 U/mL (normal < 100 U/mL). IgA and IgM normal within age adjusted range.
[15] (case report)	PA (1)	Case report	Decompensated	Evaluation: IgG, IgM, IgA. Low IgG: 230 mg/dL (normal 539–1401 mg/dL). Low IgM: 32 mg/dL (normal 47–236 mg/dL). IgA normal.
[16]	MMA (10) PA (1)	Cohort	Compensated	Evaluation: IgG, IgM, IgA, IgE. High IgG: four MMA cases. High IgM: nine MMA cases and PA case. High IgA: seven MMA cases and PA case. High IgE: five cases of MMA. Low IgE: AMM case. Rest normal.
[17]	MMA (23) PA (7)	Cross-sectional	Compensated (12) and decompensated (18)	Evaluation: IgG, IgM, IgA, IgE. None of the patients showed low levels.
[18]	MMA (21) PA (10)	Case-control	Compensated	Evaluation: IgG, IgM, IgA. Lower IgG levels than healthy controls (*p* = 0.021). No significant differences were found regarding IgA and IgM levels.
Present study	MMA (11) PA (9)	Cross-sectional	Compensated events (8), decompensated events (25)	Evaluation: IgG, IgM, IgA, IgE Low IgG in both studied groups. Tendency toward IgA and IgM higher concentrations in decompensated PD patients.

## Data Availability

The original contributions presented in the study are included in the article; further inquiries can be directed to the corresponding author due to privacy.

## Supplementary Materials

The following supporting information can be downloaded at: https://www.mdpi.com/article/10.3390/nu16111775/s1, Table S1: Age at diagnosis and age at study enrollment in patients with propionate defects.

## Abbreviations

AMM	Methylmalonic acidemia
AP	Propionic acidemia
C3	Propionylcarnitine
Cbl	Cobalamine
CD4+	Cooperative T4 lymphocytes
CD8	T8 lymphocytes
CoA	Coenzyme A
GC/MS	Gas chromatography–mass spectrometry
HPLC	High-performance liquid chromatography
IEM	Inborn error of metabolism
Ig	Immunoglobulin
MSMS	Tandem mass spectrometry
WHO	World Health Organization
