# Supplementary material for: Hypogammaglobulinemia Class G Is Present in Compensated and Decompensated Patients with Propionate Defects, Independent of Their Nutritional Status
Source: Nutrients. 2024 Jun 5;16(11):1775. doi: 10.3390/nu16111775 (PMC11174734; doi:10.3390/nu16111775)
Supplement: Supplementary file 1 [file nutrients-16-01775-s001.zip › nutrients-3019261-supplementary.pdf]

Table S1. Age at diagnosis and age at study enrollment in patients with propionate defects.

	Patient	Age at diagnosis (months)	Age at enrollment in the study (months)
**Methylmalonic acidemia**	1	0.97	3
	2	2	2
	3	2.43	1
	4	4.07	151
	5	4.27	11
	6	5	5
	7	7.2	52
	8	10.3	147
	9	15.1	84
	10	202.5	200
	11	206.3	199
**Mean age months (min-max)**		**41.8 (0.97-205.4)**	**77 (1-200)**
**Propionic acidemia**	12	0.43	11
	13	1.23	10
	14	1.43	69
	15	1.47	72
	16	1.93	34
	17	2.1	4
	18	3.1	149
	19	7	7
	20	11.13	132
**Mean age months (min-max)**		**3.3 (0.43-10.7)**	**54 (4-149)**
**Overall mean age months (min-max)**		**24.5 (0.43-206.3)**	**67 (1-200)**
